# Validation of the Antidiabetic and Hypolipidemic Effects of Hawthorn by Assessment of Gluconeogenesis and Lipogenesis Related Genes and AMP-Activated Protein Kinase Phosphorylation

**DOI:** 10.1155/2013/597067

**Published:** 2013-04-16

**Authors:** Chun-Ching Shih, Cheng-Hsiu Lin, Yih-Jiun Lin, Jin-Bin Wu

**Affiliations:** ^1^Graduate Institute of Pharmaceutical Science and Technology, College of Health Science, Central Taiwan University of Science and Technology, No. 666, Buzih Road, Beitun District, Taichung City 40601, Taiwan; ^2^Department of Internal Medicine, Fong-Yuan Hospital, Department of Health, Executive Yuan, No. 100, An-Kan Road, Fongyuan District, Taichung City 42055, Taiwan; ^3^Graduate Institute of Pharmaceutical Chemistry, China Medical University, No. 91, Hsueh-Shih Road, Taichung City 40402, Taiwan

## Abstract

Since with the increased use of antidiabetic and antihyperlipidemic effect of phytonutrients for daily supplement has gained considerable attention worldwide, we examine the effect and molecular mechanism of *Crataegus pinnatifida* Bge. var. *major* N.E. Br. (hawthorn) by quantifying the expression of hepatic gluconeogenesis and lipogenesis on diabetes and dyslipidemia in high-fat (HF)-fed C57BL/6J mice. Firstly, mice were divided randomly into two groups: the control (CON) group was fed with a low-fat diet, whereas the experimental group was fed a 45% HF diet for 8 weeks. Afterwards, the CON group was treated with vehicle, whereas the HF group was subdivided into five groups and was given orally hawthorn extract (including 0.2, 0.5, 1.0 g/kg/day extracts) or rosiglitazone (Rosi) or vehicle for 4 weeks afterward. Diabetic mice showed an increase in plasma glucose and insulin. Glucose lowering was comparable with Rosi-treated mice. This study demonstrated that hawthorn was effective in ameliorating the HF diet-induced hyperglycemia, hypertriglyceridemia and hypercholesterolaemia. Hawthorn extract significantly increases the hepatic protein contents of AMP-activated protein kinase (AMPK) phosphorylation and reduces expression of phosphenol pyruvate carboxykinase (PEPCK) and glucose production. Furthermore, hawthorn decreased in hepatic triacylglycerol and cholesterol synthesis (including sterol regulatory element binding protein-1c (SREBP-1c), fatty acid synthase (FAS), SREBP2). An increase in expressions of apoA-I gene and high-density lipoprotein cholesterol (HDL-C) was detected in HF-fed mice treated with high dose hawthorn. Our data suggest that hawthorn extract are capable of decreasing glucose production and triacylglycerol synthesis by inducing AMPK-phosphorylation and hawthorn is a candidate source of antidiabetic and antihyperlipidemic phytonutrients factors.

## 1. Introduction

Epidemiological studies have suggested that insulin resistance is associated with cardiovascular disease (CVD) risk factors, including obesity, dyslipidemia, and stroke [1]. Diabetes mellitus is a chronic metabolic disorder characterized by hyperglycemia that involves abnormalities in both insulin secretion and action at peripheral tissues. Type 2 diabetes, which accounts for more than 90%–95% of all diabetes, is characterized by the majority of metabolic defects known as insulin resistance. Insulin resistance is associated with the inability of cells to respond adequately to normal levels of insulin, occurs primarily with muscles, liver and fat tissue [2]. 

*Crataegus pinnatifida* Bge. var. *major* N.E.Br., also referred to as hawthorn, has been catalogued in a classic traditional Chinese medical book, described to improve digestion, promote blood circulation, and resolve blood stasis both in traditional and folk medicines [3]. The chemical components of hawthorn consist of triterpene acids, flavonoids, oligomeric proanthocyanidins, organic acids, and cardioactive amines [4]. Clinical evidence indicates that some hawthorn extracts may increase the exercise tolerance of patient with congestive heart failure [5]. The pharmacological effects were believed to mainly possess a number of beneficial effects such as cardiotonic [5], hypolipidemic [6, 7], and antioxidative activities [8]. Analytical results suggest that the procyanidins are the major active constituents in the Chinese hawthorn, which consists of epicatechin and procyanidin B2 [9].

Rosiglitazone has been used for the treatment of diabetes and has been used as an antidiabetic agent in humans [10] and in animals [11]. Peroxisome proliferator activated receptor (PPAR)-*γ* activators reduce circulating glucose by storing it as fat in adipocytes [12], thus leading to the reduction of lipotoxicity in liver and skeletal muscle [13]. Fenofibrate, a PPAR*α* agonist, prevents bodyweight gain by mobilizing and burning fat and downregulates lipogenic genes and eventually reduces plasma triglycerides and fatty acids [14].

The primary aim of the present study was to investigate the effects of the hawthorn-mediated glucose and lipid lowering in a diabetic and dyslipidemic mice model, HF-fed C57BL/6J mice. The second objective is to examine if hypolipidemic effects of hawthorn occur via additional mechanism not present in the rosiglitazone treated group. The C57BL/6J mice when fed an HF diet develop severe obesity, hyperglycemia, hyperlipidemia, and hyperinsulinemia [15]. Therefore, the HF-fed C57BL/6J mice model has been chosen to clarify both antidiabetic and hypolipidemic properties effects of hawthorn. Activators of PPARs are effective drugs to improve the metabolic abnormalities linking hypertriglyceridemia to diabetes, hyperglycemia, and insulin resistance. We compared the hawthorn extracts with the pharmacological profile of a PPAR*γ* activator, rosiglitazone, on serum parameters, target gene expression in HF-fed mice.

AMP-activated protein kinase (AMPK) is considered as a therapeutic target for treatment of diabetes and dyslipidemia [16]. Since AMP-activated protein kinase (AMPK) is an energy sensor and controls glucose and lipid metabolism, the effect of hawthorn on AMPK activity is investigated in HF mouse liver tissue. Phosphorylation of Thr 172 of *α* subunits is essential for AMPK activity [17]. Sterol regulatory element binding proteins (SREBPs) regulate the gene expression of enzymes involved in lipogenesis and cholesterol biosynthesis [18]. Fatty acid synthase (FAS) is the key enzyme in fatty acid synthesis [19]. As one of the possible mechanisms of action, this study also examined its effect on the expression of genes involved in antidiabetic effects and lipogenesis in the liver tissue, including sterol regulatory element binding protein-1c (SREBP-1c), FAS, and phosphoenol pyruvate carboxykinase (PEPCK).

## 2. Materials and Methods

### 2.1. Preparation of Extract and HPLC Analysis

The fruits of *C. pinnatifida* Bge. var. *major* N.E.Br. were purchased from the local markets in Taiwan in September 2008. All materials were sorted and identified by Professor Chao-Lin Kuo with voucher specimens (CMU-CP080001-CMU-CP08011) deposited in the China Medical University, Taichung, Taiwan. The dried fruits of hawthorn (20 kg) were extracted with 400 L methanol (80% in water) by 2 hr reflux. After filtration, the filtrate was concentrated under reduced pressure at 40°C and transdissolved in water and then partitioned with chloroform. The water layer was obtained and then extracted with n-butanol. The n-butanol layer was followed by n-butanol partition, and the resulting n-butanol layer was obtained for experiment. The extract yield was approximately 1.6%. The extract was diluted and adjusted and then was administrated orally to mice in a volume of 0.2, 0.5, and 1.0 g/kg bodyweight (K1: 0.2, K2: 0.5, and K3: 1.0 g/kg bodyweight), respectively. Distilled water was administered in a similar volume to control mice. The chromatography was equipped with two P200II solvent delivery units, a UV detector 5PD-6AV, and pump A is Shimadzu LC-6A and pump B is Shimadzu LC-6A. Chromatographic analysis was performed on a Shimadzu LC-6A system (Shimadzu Co., Kyoto, Japan) LiChrospher-100 RP-18 column (*φ*  4.6 × 250 mm) (Merck Millipore Co., Billerica, MA, USA). The flow rate was 0.8 mL/min, and the injection volume was 10 *μ*L. An amount of 0.05% formic acid was employed as mobile phase A, and acetonitrile was employed as mobile phase B. The gradient procedure was 0–10 min with 20%, 10–15 min with 20%–70%, 15–25 min with 70%, and 25–30 min with 70%–20%. The column temperature was 25°C, and the detection was carried out at 280 nm. The 80% MeOH-eluted fraction was chromatographed to isolated procyanidin B2 (0.15%).

### 2.2. Animal Model and Treatment

Male C57BL/6J mice (5 weeks of age) were purchased from the National Laboratory Animal Breeding and Research Center, National Science Council. These animals were housed in an air-conditioned room at 22 ± 3°C with 12 h light/dark cycle and tap water *ad libitum*. Animal experiments were performed in accordance with the guidelines of the Institutional Animal Care and Use Committee of Central Taiwan University of Science and Technology (IACUC Approval no. 97-CTUST-3). The mice were divided randomly into two groups after a 1-week acclimation period. The control (CON) group (*n* = 9) was fed low-fat diet (Diet 12450B, Research Diets, Inc., New Brunswick, NJ, USA), whereas the experimental group was fed a 45% high-fat diet (Diet 12451, Research Diets, Inc., New Brunswick, NJ, USA) for 12 weeks. The low-fat diet was composed of protein 20%, carbohydrate 70%, and fat 10%, whereas high-fat diet was composed of protein 20%, carbohydrate 35%, and fat 45% (of total energy, % kcal). After the first 8 weeks, the high-fat treated mice were further subdivided into five groups and were administrated by gavages with or without hawthorn or rosiglitazone (Rosi) for 4 weeks, while the mice were still on the high-fat diet. During the last 4 weeks, the CON and high-fat control (HF) mice were treated with vehicle only. The other groups were administrated through oral gavage 1 time per day hawthorn extract (including 0.2, 0.5, and 1.0 g/kg/day extracts) or Rosi 10 mg/kg, respectively (*n* = 9). Rosiglitazone (GlaxoSmithKline Product no. BRL49653 C) was dissolved in 1% methylcellulose. The bodyweight was measured weekly throughout the study. These dietary periods lasted for 12 weeks. The experimental diets were based on published work by Surwit et al. [20]. The compositions of the experimental diets are shown in Table 1. At the end of the experiment, the mice were killed by exsanguinations under diethyl ether anesthesia. According to the defined anatomical landmarks the liver and white adipose tissues (WATs) (including epididymal, mesenteric, and retroperitoneal WATs) were dissected. The weights of the tissues were measured. Visceral fat was defined as the sum of epididymal and retroperitoneal WATs. They were then immediately frozen using liquid nitrogen and kept at −80°C until use. The fasting collected blood was kept at room temperature for 5 min for coagulation. Then, the plasma was obtained from the coagulated blood by centrifugation at 1600 × g for 15 min at 4°C. The separation of the plasma was finished within 30 min. Aliquots of the supernatant were obtained for insulin, leptin, total cholesterol (TC), TG, and FFA assay. The plasma was immediately frozen at −80°C until use.

### 2.3. Food Intake and Bodyweight Determination

Food intake and bodyweight were measured using an electronic scale. The pellet food was firstly weighed and then placed in the cage food container; after 24 h, the remaining food was weighed. The difference represented the daily food intake. Unconsumed pellet HF food was discarded every day and fresh pellet high-fat diet was provided to ensure consistent food quality to the mice throughout the study. The food was stored at 4°C.

### 2.4. Fasting Blood Glucose and Lipid Parameters Assay

Blood samples were collected from the retroorbital sinus of fasting mice and the level of glucose was measured by the glucose oxidase method (Model 1500; Sidekick Glucose Analyzer; YSI Incorporated, Yellow Springs, USA). The concentrations of triglyceride (TG), total cholesterol (TC), free fatty acid (FFA), high-density lipoprotein (HDL-C), and low-density lipoprotein (LDL-C) were measured using commercial assay kits according to the manufacturer's directions (Triglycerides-E test, Cholesterol-E test, and FFA-C test, Wako Pure Chemical, Osaka, Japan; HDL-C-test and LDL-C-test, Roche Diagnostics GmbH, Indianapolis, USA).

### 2.5. Adipocytokine Levels Assay

The levels of insulin and leptin were measured by ELISA using a commercial assay kit according to manufacturer's directions (mouse insulin ELISA kit, Shibayagi, Gunma, Japan, and mouse leptin ELISA kit, Morinaga, Yokohama, Japan).

### 2.6. Histology Analysis of Epididymal WAT

Small pieces of epididymal WAT were fixed with formalin (200 g/kg) neutral buffered solution and embedded in paraffin. Sections (8 *µ*m) were cut and stained with hematoxylin and eosin. For microscopic examination, a microscope (Leica, DM2500) was used, and the images were taken using a Leica Digital camera (DFC-425-C) at 10 (ocular) × 40 (object lens) magnification.

### 2.7. Relative Quantization of mRNA Indicating Gene Expression

Total RNA from the epididymal WAT, skeletal muscle, and liver was isolated with a Trizol Reagent (Molecular Research Center, Inc., Cincinnati, OH, USA) according to the manufacturer's directions. The integrity of the extracted total RNA was examined by 2% agarose gel electrophoresis, and the RNA concentration was determined by the ultraviolet (UV) light absorbency at 260 nm and 280 nm (Spectrophotometer U-2800A, Hitachi). The quality of the RNA was confirmed by ethidium bromide staining of 18S and 28S ribosomal RNA after electrophoresis on 2% agarose gel containing 6% formaldehyde. Total RNA (1 *μ*g) was reverse-transcribed to cDNA in a reaction mixture containing buffer, 2.5 mM dNTP (Gibco-BRL, Grand Island, NY), 1 mM of the oligo (dT) primer, 50 mM dithiothreitol, 40 U Rnase inhibitor (Gibco-BRL, Grand Island, NY), and 5 *μ*L Moloney murine leukemia virus reverse transcriptase (Epicentre, USA) at 37°C for 1 h and then heated at 90°C for 5 min to terminate the reaction. The polymerase chain reaction (PCR) was performed in a final 25 *μ*L containing 1 U Blend Taq-Plus (Toyobo Co., Osaka, Japan), 1 *μ*L of the RT first-strand cDNA product, 10 *μ*M of each forward (F) and reverse (R) primer, 75 mM Tris-HCl (pH 8.3) containing 1 mg/L Tween 20, 2.5 mM dNTP, and 2 mM MgCl_2_. Preliminary experiments were carried out with various cycles to determine the nonsaturating conditions of the PCR amplification for all the genes studied. The primers are shown in Table 2. The products were run on 2% agarose gels and stained with ethidium bromide. The relative density of the band was evaluated using AlphaDigiDoc 1201 software (Alpha Innotech Co., San Leandro, CA, USA). All the measured PCR products were normalized to the amount of cDNA of GAPDH in each sample.

### 2.8. Western Immunoblotting Analysis of Phospho-AMPK (Thr172) Proteins

Protein extractions and immunoblots for the determination of AMPK phosphorylation were carried out on frozen liver tissue from mice according to a previous report [21]. Briefly, liver samples (0.1 g) were powdered under liquid nitrogen and homogenized for 20 s in 500 *μ*L buffer containing 20 mM Tris-HCl (pH 7.4 at 4°C ), 2% SDS, 5 mM EDTA, 5 mM EGTA, 1 mM DTT, 100 mM NaF, 2 mM sodium vanadate, 0.5 mM phenylmethylsulfonyl fluoride, 10 *μ*g/mL leupeptin, and 10 *μ*L/mL pepstatin. 40 *μ*g of each homogenate was mixed with an equal amount of 2 × standard SDS sample loading buffer containing 125 mM Tris-HCl (pH 6.8), 4% SDS, 20% glycerol, 10% *β*-mercaptoethanol, and 0.25% bromophenol blue and boiled for 10 min before electrophoresis. Proteins were separated by 12% SDS-PAGE according to the method of Laemmli [22] and transferred by electroblotting onto PolyScreen PVDF transfer membrane (NEN) using semidry transfer cell (Bio-Rad) according to the manufacturer's manual. The membrane was then treated sequentially with blocking solution (phosphate-buffered saline (PBS) containing 5% nonfat skim milk), with appropriate dilution of anti-phospho-AMPK*α* (Thr 172) antibody (Abcam Inc., USA) and with anti-(G6PD) G6PD (glucose 6 phosphate dehydrogenase antibody; Abcam Inc., USA) conjugated to peroxidase (Zymed). Finally, the membrane was soaked in a chromogen/substrate solution (TMB single solution; Zymed) for color development.

### 2.9. Oral Glucose Tolerance Test (OGTT)

The ICR normal mice (*n* = 5) were fasted for 15–18 h but were allowed access to 0.2 g/kg, 1.0 g/kg, and 2.0 g/kg extracts of hawthorn or an equivalent amount of normal saline was given orally 30 min before an oral glucose load (1 g/kg bodyweight). Blood samples were collected at the time of the glucose administration (0) and every 30 minutes until 3 hours after glucose administration to determine the levels of glucose.

### 2.10. Statistical Analysis

The results were expressed as mean ± S.E. The statistical significance was evaluated by one-way analysis of variance (ANOVA) followed by Dunnett's post hoc test using SPSS/11.5 software. Values were considered statistically significant at *P* < 0.05.

## 3. Results

### 3.1. Bodyweight, Bodyweight Gain, Food Intake, and Tissue Weight

All group mice started with similar mean bodyweights (18.33 ± 0.18 g). As shown in Table 3, at week 12, the bodyweight of all the high-fat diet treated groups is significantly greater than the CON group (*P* < 0.05), and K3-treated groups significantly decreased the bodyweight compared with the HF group (*P* < 0.01). The K2- and K3-treated groups showed a significant reduction in bodyweight gain over 4 weeks compared with the HF group (*P* < 0.001, *P* < 0.001, resp.). K3 had a significant suppressive effect on the 4-week cumulative food intake compared with the HF group (*P* < 0.01). At week 12, the weights of absolute adipose tissue were markedly greater in the HF group than in the CON group (epididymal WAT + 68.9% (*P* < 0.05), retroperitoneal WAT +126.5% (*P* < 0.05), and visceral fat + 75.8% (*P* < 0.05)). Treatment with K3 significantly decreased the weights of absolute epididymal, mesenteric, and retroperitoneal WATs, visceral fat, and liver tissue compared with the HF group (*P* < 0.05, *P* < 0.05, *P* < 0.05, *P* < 0.05, *P* < 0.05, resp.) (Table 3).

### 3.2. Plasma Glucose and Lipid Levels

**Figure 1:**
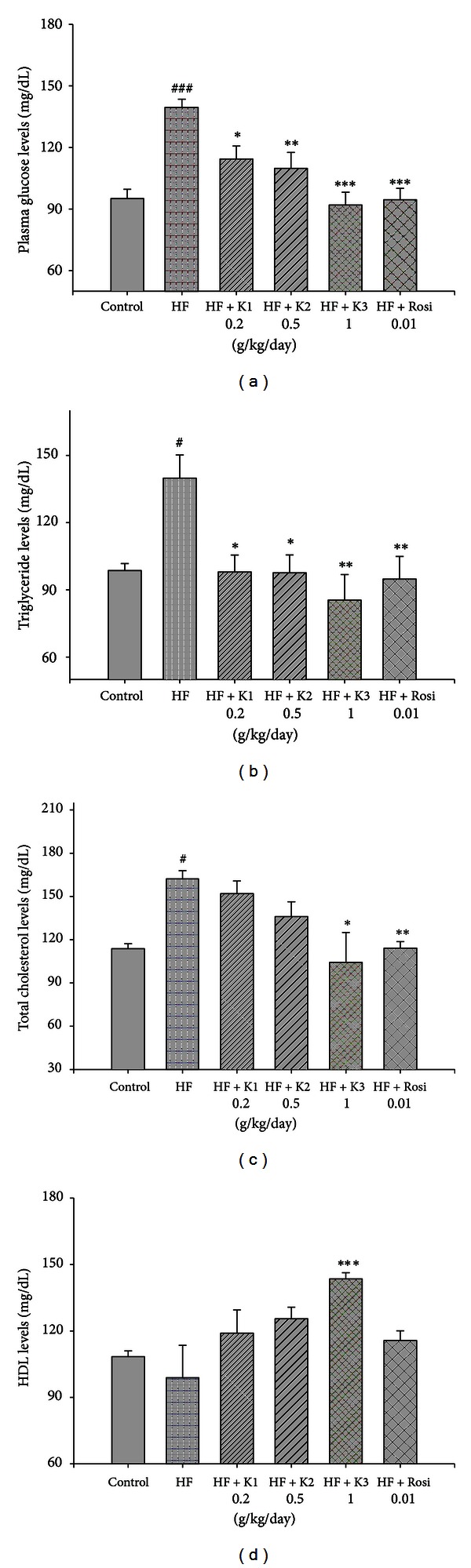
Effects of extract of hawthorn on (a) plasma glucose, (b) triglyceride, (c) total cholesterol, and (d) HDL levels at week 12. Mice were fed 45% high-fat diet (HF) or low-fat diet (CON) for 12 weeks. At 8 weeks following HF, the HF mice were treated with vehicle (water; p.o.) or extracts of hawthornor rosiglitazone (p.o.) accompanied with HF diet for 4 weeks. All values are means ± S.E. (*n* = 9). ^#^ *P* < 0.05 compared with the control (CON) group; *P < 0.05, **P < 0.01, ***P < 0.001 compared with the high-fat + vehicle (distilled water) (HF) group by ANOVA.

At week 8, the glucose, TG, and TC levels of the HF group were significantly greater than the CON group (126.2 ± 3.4 versus 91.6 ± 10.8 mg/dL, 92.7 ± 5.6 versus 73.5 ± 4.8 mg/dL, and 118.1 ± 2.7 versus 81.4 ± 4.4 mg/dL, resp.) (*P* < 0.05, *P* < 0.05, *P* < 0.001, resp.). As time passed, the hypercholesterolemic phenomenon was evident for the HF diet. As shown in Figure 1, at week 12, the levels of glucose, TG, and TC were significantly greater in the HF group than in the CON group (*P* < 0.001, *P* < 0.05, and *P* < 0.05, resp.) (Figures 1(a), 1(b), and 1(c)). Following treatment, all the hawthorn- and Rosi-treated groups showed a significant reduction in glucose and TG levels compared with the HF group (Figures 1(a) and 1(b)). The K3- and Rosi-treated groups suppressed the high-fat diet-induced increases in the TC concentrations (Figure 1(c)). There was no significant difference in HDL levels between the CON group and the HF group. The K3-treated groups increased the HDL levels compared with the HF group (*P* < 0.001) (Figure 1(d)). As shown in Table 3, at week 12, the levels of FFA and LDL were greater in the HF group than in the CON group (*P* < 0.05, *P* < 0.001, resp.). The K2- and K3- and Rosi-treated groups suppressed the high-fat diet-induced increases in the concentrations of FFA. The K3-treated groups suppressed the high-fat diet-induced increases in the concentrations of LDL (*P* < 0.05).

### 3.3. Leptin and Insulin Concentration

At week 12, the concentrations of leptin and insulin were greater in the HF group than in the CON group (*P* < 0.01, *P* < 0.05, resp.). The K3- and Rosi-treated groups significantly decreased the levels of leptin and insulin compared with the HF group (Table 3).

### 3.4. Homeostasis Model Assessment for Insulin Resistance

At week 12, the levels of insulin resistance were significantly greater in the HF group than in the CON group (*P* < 0.01). Following treatment, K2-, K3-, and Rosi-treated groups showed a significant reduction in insulin resistance compared with the HF group (*P* < 0.05, *P* < 0.01, *P* < 0.01, resp.) (Table 3).

### 3.5. Expressions of PPAR*γ* and Glucose Transporter 4 (GLUT4)

At week 12, no significant difference was observed in adipose PPAR*γ* expression of mRNA between the HF group and the CON group. After treatment, the mRNA level of PPAR*γ* was greater in the K3- and Rosi-treated groups than in the HF group (Table 4). At week 12, the mRNA levels of GLUT4 were lower in the HF group than in the CON group. All the hawthorn-treated groups and Rosi-treated groups significantly increased the skeletal muscular expression of GLUT4 compared with the HF group (Table 4).

### 3.6. Epididymal WAT Histology

**Figure 2:**
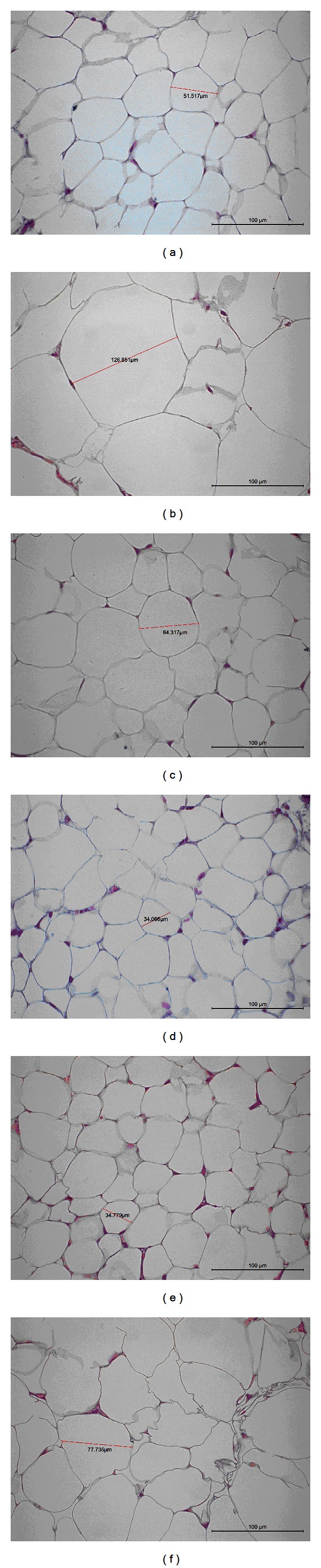
Histology of the epididymal white adipose tissue (WAT) of mice in the (a) low-fat (LF), (b) high-fat (HF), (c) HF + K1, (d) HF + K2, (e) HF + K3, or (f) HF + Rosi groups. Each image presented is typical and representative of nine mice. Magnification: 10 (ocular) × 40 (object lens). K1: 0.2, K2: 0.5, and K3: 1.0 g/kg bodyweight) extracts of hawthorn; Rosi: rosiglitazone (0.01 g/kg bodyweight).

As shown in Figure 2, feeding the HF diet induced hypertrophy of the adipocytes compared with the CON group in epididymal WAT. Afterwards, treatment with hawthorn (K2 and K3) decreased the hypertrophy compared with the HF group. The results obtained from the other mice were similar to those shown in Figure 2.

### 3.7. Expressions of PEPCK, SREBP1c, Fatty Acid Synthase (FAS), PPAR*α*, Apolipoprotein C-III (apo C-III), Apolipoprotein A-I (apo A-I), and SREBP-2 in Liver Tissue

**Figure 3:**
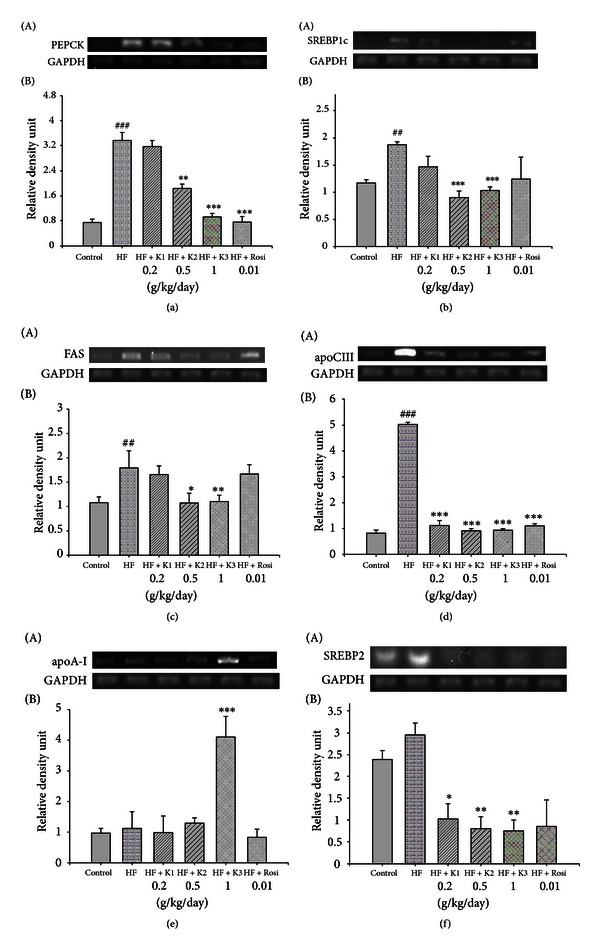
Semiquantitive RT-PCR analysis on (a) PEPCK, (b) SREBP1c, (c) FAS, (d) apo C-III, (e) apoA-I, and (f) SREBP2 mRNA expression in liver tissue of the mice by oral gavage extracts of hawthorn for 4 weeks. All values are means ± S.E. (*n* = 9). ^##^ *P* < 0.01, ^###^ *P* < 0.001 compared with the control (CON) group; *P < 0.05, **P < 0.01, ***P < 0.001 compared with the high-fat + vehicle (distilled water) (HF) group. K1, K2, K3, extracts of hawthorn.

As shown in Figure 3, at week12, the mRNA levels of PEPCK, SREBP1c, and FAS were greater in the HF group than in the CON group (*P* < 0.001, *P* < 0.01, *P* < 0.01, resp.). The K2- and K3-treated groups significantly decreased the mRNA level of FAS, PEPCK, and SREBP1c compared with the HF group. At week 12, the mRNA levels of apo C-III were greater in the HF group than in the CON group (*P* < 0.001). Treatment with K1, K2, K3, and Rosi significantly decreased the expression of apo C-III (*P* < 0.001, *P* < 0.001, *P* < 0.001, *P* < 0.001, resp.). At week 12, there was no significant difference in the expressions of apo A-I between the HF group and the CON group. Following treatment, the mRNA level of apoA-I was greater in the K3-treated group than in the HF group (*P* < 0.001). All the hawthorn-treated groups significantly decreased the mRNA level of SREBP-2 compared with the HF group (Figure 3). At week 12, the mRNA level of PPAR*α* did not differ between the HF group and the CON group (Table 4). After treatment, K3 significantly increased the mRNA level of PPAR*α* compared with the HF group (*P* < 0.05) (Table 4).

### 3.8. Western Immunoblotting Contents of Phospho-AMPK (Thr172) Protein

**Figure 4:**
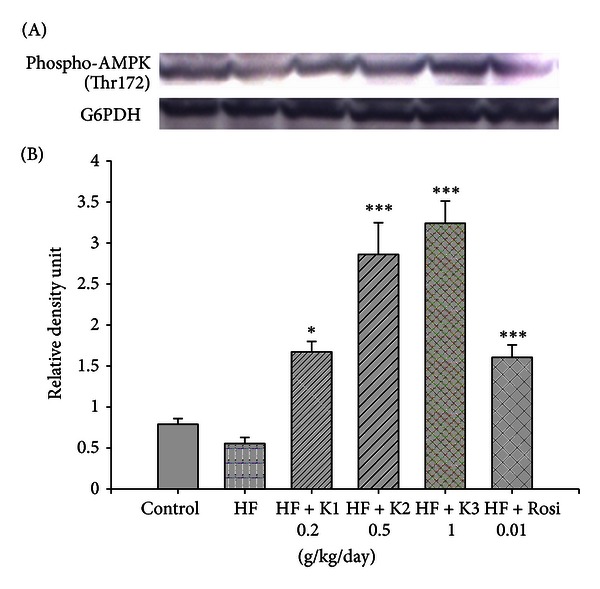
The phospho-AMPK (Thr172) protein contents in liver tissue of the mice by oral gavage extracts of hawthorn for 4 weeks. Protein was separated by 12% SDS-PAGE detected by Western blot. All values are means ± S.E. (*n* = 9). *P < 0.05, ***P < 0.001 compared with the high-fat + vehicle (distilled water) (HF) group by ANOVA. K1, K2, K3, extracts of hawthorn.

At week 12, no significant difference was observed in phospho-AMPK protein between the HF group and the CON group. Following treatment, the contents of phospho-AMPK protein were increased in the K1-, K2-, K3-, and Rosi-treated groups compared with the HF group in liver tissue (*P* < 0.05, *P* < 0.001, *P* < 0.001, *P* < 0.001, resp.) (Figure 4).

### 3.9. Oral Glucose Tolerance Test

**Figure 5:**
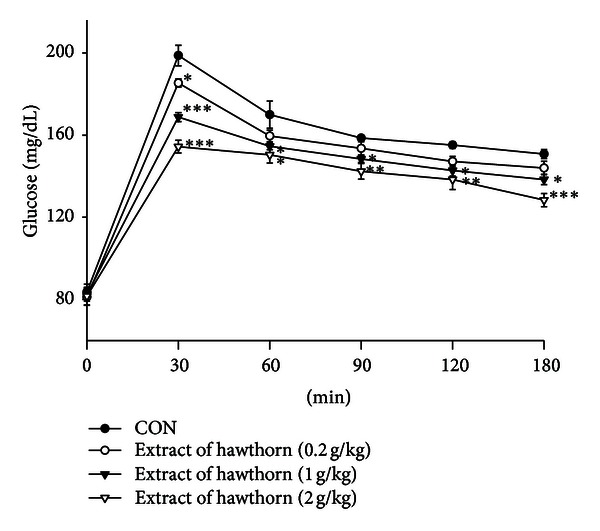
Effects of extract of hawthorn on oral glucose tolerance in normal mice. Animals in all groups received oral glucose 30 minutes after the extract administration. Blood samples were collected and centrifuged at 3000 rpm for 10 minutes. Each point is the mean ± S.E. of 5 separate mice. *P < 0.05, **P < 0.01, ***P < 0.001 are significantly different compared with the control group in the same time by ANOVA.

As shown in Figure 5, following treatment with 0.2 g/kg extract of hawthorn, the increased blood glucose levels were significantly suppressed after 30 min. Following treatment with hawthorn extract (1.0 and 2.0 g/kg), the blood glucose levels were significantly decreased at 30, 60, 90, 120, and 180 min as compared with the control (Figure 5).

## 4. Discussion

Agents suppressing the blood glucose and triglyceride by enhancing AMPK activation and GLUT4 while reducing PEPCK and hepatic lipogenesis may constitute a useful mechanic approach to both antidiabetic and hypolipidemic therapies. Many antidiabetic and hypolipidemic agents have been developed, but unfavorable side effects are serious problem. Therefore, there is need for more effective antidiabetic and hypolipidemic agents. This study was firstly undertaken to clarify the antidiabetic effect of hawthorn and compare blood glucose levels with the marketed drug, rosiglitazone, which lowers plasma glucose primarily by insulin sensitization. In this study, similar to Chaput et al. [23] results, rosiglitazone regarding lowered blood glucose and serum triglycerides, whereas increased bodyweight gain. In this study, we demonstrated that hawthorn extract effectively controlled hyperglycemia by significantly reducing blood glucose levels in C57BL/6J mice on an HF diet. Moreover, FFA is reported to be one of the candidate molecules in causing insulin resistance [24] and HOMA-IR index lowering effects, suggesting that hawthorn at middle and high dose could improve insulin resistance.

In this study, similar to the results obtained by Kuo et al. [25], significant reduction of WATs mass and bodyweight and improved dyslipidemia were seen. To understand the mechanism of fatty acid and triglyceride lowering as well as glucose and insulin lowering, mRNA of key enzyme of glucose production in the liver, PEPCK, and key transcription factor that induces lipogenic genes, SREBP1c and FAS, were quantitated.

PEPCK is a key enzyme of gluconeogenesis. In diabetic animal models, upregulation of PEPCK expression is noticed [26], suggesting that the restoration of elevated PEPCK expression can be considered a good therapeutic target for diabetes therapy [27]. AMPK activation is known to reduce hepatic gluconeogenesis and PEPCK expression, resulting in reduced glucose levels [26]. PEPCK expressions were significantly decreased in the liver of K2- and K3-treated mice. This could be due to increased contents of phosphorylation AMPK. The lowered blood glucose in hawthorn-treated mice may be caused by decreased gluconeogenesis which is controlled by PEPCK. The decreases in PEPCK expressions might indicate a decrease in glyconeogenesis in the liver. This might also indicate that hawthorn has the ability to improve hyperglycemia through hawthorn-stimulated AMPK activities in glyconeogenesis. Thus, it resulted in downregulation of PEPCK gene expression. This indicates a potential role for hawthorn in improving insulin resistance.

AMPK is a critical node linking signaling and lipid metabolism and a positive regulator of insulin resistance [28]. The downregulation of AMPK activity, which is indicated by its phosphorylation level, reflects potential negative effects of lipid concentrations on insulin signaling [29]. Hawthorn significantly increased phosphorylation of AMPK, and not only improved lipid metabolism but also improved glucose uptake in skeletal muscle. PPAR*γ* has been shown to increase GLUT4 gene expression or plasma membrane translocation, contributing to improving glucose homeostasis [30, 31]. Our study showed that hawthorn increased GLUT4 expression in skeletal muscle, suggesting that hawthorn on glucose homeostasis caused an improvement in muscle glucose uptake.

The second objective of this study was to look into the mechanism of hypolipidemic effect of hawthorn. Following treatment with hawthorn, triglycerides and free fatty acids lowering occurred as a downregulation of the transcription factor SREBP1c, which upregulates a number of lipogenic genes [32]. In PPAR*α*-deficient mice, dysregulation of SREBP-mediated lipogenic genes was noticed [33], suggesting the role of PPAR*α* in SREBP-mediated regulation of lipogenic genes in mouse model of dyslipidemia [14], in the present study, further confirming hawthorn's lipid-lowering effects via downregulation of genes involved in lipid synthesis. In this study, hawthorn significantly increased the mRNA expression levels of fatty acid oxidative enzyme (PPAR*α*), whereas it suppressed the expression of SREBP1c (involved in triacylglycerol synthesis) and SREBP2 (involved in total cholesterol synthesis) in the liver of HF-fed mice. Treatment with hawthorn resulted in the reduction of FAS mRNA level, suggesting that hawthorn would at least downregulate the gene expression of the enzymes through a reduction in the SREBP1c mRNA level. Moreover, the glucose-induced FAS mRNA level was also downregulated by AMPK [34, 35], suggesting that hawthorn might accelerate direct or leptin-mediated AMPK activation. Metformin is reported to downregulate the SREBP1c expression, thereby decreasing the FAS mRNA level through AMPK activation [36]. Thus, hawthorn possibly inactivated these enzymes and/or down regulate the SREBP1c expression, thereby reducing the FAS mRNA level through AMPK activation.

In this study, hawthorn is proved to improve lipid metabolism through the regulation of PPARs expressions. PPAR*α* ligands also decrease the expression of apo C-III, resulting in hypotriglyceridemic effect [37]. Moreover, the increased activity of PPAR*α* and gene expression in the liver plays an essential role in regulating fatty acid oxidation [38]. Thus, our results suggest that hawthorn improves plasma lipid profiles by stimulating fatty acid oxidation through PPAR*α*-mediated pathways.

Additional mechanism that could play a role in hypolipidemic effect of hawthorn was also examined. Our result regarding hawthorn lowering the levels of cholesterol is consistent with previous studies [6, 25]. SREBP2 plays a major role in the regulation of cholesterol synthesis [39], suggesting that the potential mechanism of hawthorn is associated with SREPB2 on the inhibitory action of cholesterol synthesis. Additionally, in this study, hawthorn increased HDL-C levels accompanied with the increasing apo A-I expression. PPAR*α* ligands are used widely to lower serum TG whereas they increase HDL-C in patients with obesity and dyslipidemia [40]. Since both apo A-I is reported to be the primary protein of HDL-C [41] and apo A-I is synthesized by liver cell [42], the observed increased HDL-C levels are possibly mediated by increased hepatic apo A-I production.

In this study, hawthorn (at middle and high doses) significantly reduced bodyweight gain. The bodyweight gain is known to result from an imbalance between energy intake and energy expenditure [43]. The WAT is reported to be the tissue associated with energy storage [44]. Therefore, the decreased weight gain may be partly associated with the decreased total energy storage, furthermore, partly because hawthorn (at middle and high doses) reduced food intake. Most importantly, it is noteworthy finding that treatment with hawthorn markedly increased the phosphorylation of AMPK. AMPK also plays an important role in the regulation of whole-body energy metabolism.

Analysis of WAT histology shows that hawthorn decreasing the adipose tissue mass by decreasing the number of large adipocytes and increasing the number of small adipocytes (Figure 2). Due to tha fact that lipids that accumulate in adipose tissue are largely from circulating triacylglycerols [45], adipocytes size seems to be influenced by circulating triglycerides. Since liver is a major target tissue for lipid and lipoprotein metabolism, hawthorn might mobilize fat from adipose tissue by increasing hepatic fat catabolism. This is supported by our results showing that the decreased hepatic triacylglycerol synthesis might effectively decrease adipose tissue mass.

In conclusion, since AMPK is a major cellular regulator of glucose and lipid metabolism, hawthorn increases insulin sensitivity associated with the phosphorylation and activation of AMPK. Furthermore, AMPK activation leads to decreased hepatic glucose production, resulting in reduced glucose level in HF-fed mice. Moreover, by increasing the phosphorylation of AMPK in liver tissue, hawthorn should decrease hepatic fatty acid synthesis and, on the other hand, increase fatty acid oxidation, which, in turn, contributed to the lowering of circulating triglycerides. Theoretically activation of AMPK provides an explanation for many of the pleiotropic beneficial effects of hawthorn. Our findings demonstrated that hawthorn had the therapeutic potential for the protection against diabetes and hyperlipidemia.

## Figures and Tables

**Table 1:** Composition of the high- and low-fat diets (kcal).

Ingredient	Low-fat	High-fat
Casein	800	800
L-Cystine	12	12

Corn starch	1260	291
Maltodextrin 10	140	400
Sucrose	1400	691
Cellulose, BW200	0	0

Soybean oil	225	225
Lard*	180	1598

Mineral Mix S10026	0	0
Dicalcium carbonate	0	0
Calcium carbonate	0	0
Potassium citrate, 1 H_2_O	0	0

Vitamin Mix V10001	40	40
Choline bitartrate	0	0

FD&C Yellow Dye no. 5	0	
FD&C Red Dye no. 40		0
FD&C Blue Dye no. 1		

Total	4057	4057

**Table 2:** Primers used in this study.

Gene	Accession number	Forward primer and reverse primer	PCR product (bp)	Annealing temperature (°C)
White adipose tissue				
PPAR*γ*	NM_013124	F: CATGCTTGTGAAGGATGCAAG R: TTCTGAAACCGACAGTACTGACAT	190	55
Skeletal muscle				
Glut4	M25482	F: ACTGGCGCTTTCACTGAACT R: CGAGGCAAGGCTAGATTTTG	106	56
Liver				
PPAR*α*	NM_011144	F: ACCTCTGTTCATGTCAGACC R: ATAACCACAGACCAACCAAG	352	55
apoA-I	NM_009692.3	F: ACATATATAGACCAGGGAAGAA R: AAACTGGGACACATAGTCTCT	246	50.5
apoC-III	NM_023114.3	F: CAGTTTTATCCCTAGAAGCA R: TCTCACGACTCAATAGCTG	349	47
FAS	NM_007988	F: TGGAAAGATAACTGGGTGAC R: TGCTGTCGTCTGTAGTCTTG	240	50
PEPCK	NM_011044.2	F:CTACAACTTCGGCAAATACC R:TCCAGATACCTGTCGATCTC	330	52
SREBP1c	NM_011480	F: GGCTGTTGTCTACCATAAGC R: AGGAAGAAACGTGTCAAGAA	219	50
SREBP2	AF289715.2	F: ATATCATTGAAAAGCGCTAC R: ATTTTCAAGTCCACATCACT	256	47
GAPDH	NM_031144	F: TGTGTCCGTCGTGGATCTGA R: CCTGCTTCACCACCTTCTTGA	99	55

**Table 3:** Absolute tissue weight, 4-week cumulative food intake, and blood profiles.

Parameter	CON	HF	HF+K1	HF+K2	HF+K3	HF+Rosi
	−	−	0.2^a^	0.5^a^	1.0^a^	0.01^a^
Final body weight (g)	24.82 ± 0.64	27.38 ± 0.58^#^	25.74 ± 0.77	25.60 ± 0.58	23.73 ± 0.66**	26.25 ± 1.03
Body weight gain (g)	0.55 ± 0.16	0.88 ± 0.46	−0.37 ± 0.18	−1.33 ± 0.59***	−1.86 ± 0.44***	0.53 ± 0.18
Food intake (kcal/mouse)	256.32 ± 9.51	279.35 ± 11.24	275.12 ± 5.10	268.46 ± 6.36	233.98 ± 5.35**	284.05 ± 8.58
Absolute tissue weight (g)						
EWAT	0.370 ± 0.034	0.625 ± 0.093^#^	0.609 ± 0.079	0.461 ± 0.046	0.364 ± 0.027*	0.368 ± 0.095*
MWAT	0.276 ± 0.018	0.343 ± 0.033	0.301 ± 0.028	0.321 ± 0.024	0.241 ± 0.019*	0.302 ± 0.032
RWAT	0.098 ± 0.011	0.222 ± 0.049^#^	0.206 ± 0.036	0.153 ± 0.023	0.104 ± 0.013*	0.178 ± 0.037
Visceral fat	0.467 ± 0.043	0.821 ± 0.103^#^	0.815 ± 0.07	0.613 ± 0.068	0.468 ± 0.037*	0.495 ± 0.130*
BAT	0.0382 ± 0.0023	0.0390 ± 0.0046	0.0323 ± 0.0019	0.0355 ± 0.0019	0.0327 ± 0.0018	0.0500 ± 0.0040
Liver (g)	0.755 ± 0.028	0.798 ± 0.029	0.786 ± 0.023	0.719 ± 0.028	0.690 ± 0.036*	0.764 ± 0.023
Blood profiles						
FFA (meq/L)	1.473 ± 0.105	2.080 ± 0.181^#^	1.720 ± 0.267	1.498 ± 0.166*	1.487 ± 0.151*	1.305 ± 0.060*
LDL (mg/dL)	69.6 ± 5.9	124.7 ± 13.4^###^	92.8 ± 6.5	107.0 ± 7.2	88.2 ± 8.6*	134.3 ± 6.4
Leptin (*μ*g/mL)	1.619 ± 0.361	6.579 ± 1.442^##^	3.802 ± 1.197	3.301 ± 0.820	2.528 ± 0.479*	2.195 ± 0.527*
Insulin (*μ*g/L)	0.633 ± 0.091	0.929 ± 0.021^#^	0.865 ± 0.041	0.758 ± 0.087	0.581 ± 0.064*	0.533 ± 0.020*
Insulin resistance	3.27 ± 0.35	7.03 ± 0.81^##^	5.37 ± 0.64	4.52 ± 0.47*	2.90 ± 0.30**	2.73 ± 0.29**

All values are means ± S.E. (*n* = 9). ^#^ *P* < 0.05, ^##^ *P* < 0.01, ^###^ *P* < 0.001 compared with the control (CON) group; *P < 0.05, **P < 0.01, ***P < 0.001 compared with the high-fat + vehicle (distilled water) (HF) group. K1, K2, K3, extracts of hawthorn. EWAT: epididymal white adipose tissue; RWAT: retroperitoneal white adipose tissue; MWAT: mesenteric white adipose tissue; visceral fat: EWAT + RWAT; FFA: plasma free fatty acid. The homeostasis model assessment for insulin resistance scores, according to the following formula: (milligrams of glucose per deciliter × microunits of insulin per milliliter) ÷ 405. Higher numbers indicate greater insulin resistance.

^a^Dose (g/kg/day).

**Table 4:** Semiquantitive RT-PCR analysis for mRNA expression in liver, adipose tissue, and skeletal muscle.

Parameter	CON	HF	HF + K1	HF + K2	HF + K3	HF + Rosi
	—	—	0.2^a^	0.5^a^	1.0^a^	0.01^a^
Liver						
PPAR*α*	2.19 ± 0.69	1.60 ± 0.17	2.08 ± 0.43	2.38 ± 0.25	3.82 ± 0.75*	—
White adipose tissue						
PPAR*γ*	0.40 ± 0.09	0.42 ± 0.07	1.32 ± 0.67	3.45 ± 2.63	5.23 ± 2.28*	5.99 ± 0.82***
Skeletal muscle						
GLUT4	0.40 ± 0.10	0.20 ± 0.06^#^	0.63 ± 0.17***	0.86 ± 0.14***	0.99 ± 0.03***	1.09 ± 0.03***

All values are means ± S.E. (*n* = 9). ^#^ *P* < 0.05, compared with the control (CON) group; *P < 0.05, **P < 0.01, ***P < 0.001 compared with the high-fat + vehicle (distilled water) (HF) group. Total RNA (1 *μ*g) isolated from tissue was reverse-transcribed by MMLV-RT; 10 *μ*L of RT products was used as templates for PCR. Signals were quantitated by image analysis; each value was normalized by GAPDH. K1, K2, K3, extracts of hawthorn.

^a^Dose (g/kg/day).

## Abbreviations

apo A-I: apolipoprotein A-I
apo C-III: apolipoprotein C-III
BAT: brown adipose tissue
CON: control
FAS: fatty acid synthase
FFA: free fatty acid
HDL: high-density lipoprotein
HDL-C: high-density lipoprotein cholesterol
HF: high-fat control
LDL: low-density lipoprotein
LDL-C: low-density lipoprotein cholesterol
PEPCK: phosphoenolpyruvate carboxykinase
PPAR*α*: peroxisome proliferator-activated receptor *α*
PPAR*γ*: peroxisome proliferator-activated receptor *γ*
PPARs: peroxisomal proliferator-activated receptors
Rosi: rosiglitazone
RT-PCR: reverse transcription-polymerase chain reaction
SREBPs: sterol regulatory element binding proteins
SREBP-1c: sterol regulatory element binding protein 1c
SREBP-2: sterol regulatory element binding protein-2
TC: total cholesterol
TG: triglyceride
WATs: white adipose tissues.
